# Long term for patients with futile endovascular reperfusion after stroke

**DOI:** 10.1111/cns.14588

**Published:** 2024-03-12

**Authors:** Mengke Zhang, Ruiwen Che, Jiali Xu, Wenting Guo, Xi Chen, Wenbo Zhao, Changhong Ren, Milan Jia, Xunming Ji

**Affiliations:** ^1^ Department of Neurology, Xuanwu Hospital Capital Medical University Beijing China; ^2^ Department of Neurology, Beijing ShiJiTan Hospital Capital Medical University Beijing China; ^3^ Department of Rehabilitation Medicine, Beijing ShiJiTan Hospital Capital Medical University Beijing China; ^4^ Department of Neurology Zhejiang Provincial People's Hospital Zhejiang China; ^5^ Beijing Key Laboratory of Hypoxia Conditioning Translational Medicine, Xuanwu Hospital Capital Medical University Beijing China; ^6^ Department of Neurosurgery, Xuanwu Hospital Capital Medical University Beijing China

**Keywords:** endovascular therapy, futile recanalization, long‐term outcome, stroke

## Abstract

**Aims:**

With the progress of thrombectomy technology, the vascular recanalization rate of patients with stroke has been continuously improved, but the proportion of futile recanalization (FR) is still quite a few. The long‐term prognosis and survival of patients with FR and its influencing factors remain unclear.

**Methods:**

Consecutive patients who received endovascular treatment (EVT) for ischemic stroke were enrolled between 2013 and 2021 from a single‐center prospectively registry study. We evaluated the long‐term outcome of these patients by Kaplan–Meier survival analysis, and the multivariable logistic regression curve was performed to analyze influencing factors.

**Results:**

Among 458 patients with FR, 56.4% of patients survived at 1 year, and 50.4% at 2 years. In the multivariate regression analysis, age, premorbid modified Rankin Scale (mRS), National Institutes of Health Stroke Scale (NIHSS), posterior circulation infarct, general anesthesia, symptomatic intracerebral hemorrhage (sICH), and decompressive craniectomy were found to be related to unfavorable outcomes in long‐term. Age, premorbid mRS, NIHSS, general anesthesia, and sICH were predictors of long‐term mortality.

**Conclusions:**

Futile recanalization accounts for a large proportion of stroke patients after thrombectomy. This study on the long‐term prognosis of such patients is beneficial to the formulation of treatment plans and the prediction of therapeutic effects.

## Graphical abstract

Futile recanalization accounts for a large proportion of stroke patients after thrombectomy. We evaluated the long‐term outcome of these patients by Kaplan–Meier survival analysis, and the multivariable logistic regression curve was performed to analyze influencing factors, which is beneficial to the formulation of treatment plans and the prediction of therapeutic effects.

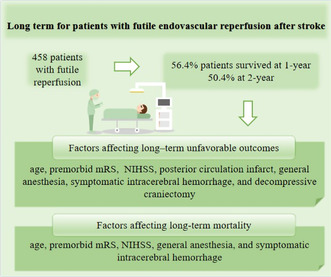

## 1 INTRODUCTION

Acute ischemic stroke (AIS) accounts for about 70% of stroke types in China, and the lifetime risk of patients is 39.3%, ranking first in the world.^1^ Endovascular treatment (EVT) has become the standard treatment of stroke due to large vessel occlusion.^2^, ^3^ Although mechanical thrombectomy (MT) affords 71%–84% recanalization rates,^4^, ^5^ more than 50% of patients do not have a favorable clinical outcome,^6^ which means futile recanalization (FR). In various studies, FR was defined as failure to achieve functional independence at 90 days despite recanalization of occluded vessels after EVT, which reached modified thrombolysis in cerebral infarction (mTICI) grade 2b or 3.

FR has been paid more and more attention by clinicians recently on account of successful recanalization is not the ultimate goal, but how to achieve a significant improvement in the prognosis of patients. Researchers have investigated the mechanisms and predictors giving rise to FR, in order to explore the effective intervention approaches to reduce the incidence of FR. There has not been any study focusing on long‐term outcomes of patients with FR yet. Thus, our study aims to analyze the long‐term prognosis and its influencing factors in patients with FR.

## 2 MATERIALS AND METHODS

### 2.1 Study design and patient selection

This observational cohort based on a prospective registry at the Xuanwu Stroke Center program includes data from consecutive AIS patients undergoing recanalization therapy (i.e., intravenous thrombolysis and EVT) since 2012. The research ethics committee at Xuanwu Hospital of Capital Medical University approved the design of this study. Verbal and written informed consent were obtained from patients or legal representatives. EVT has been conducted in the light of the guidelines. All potential patients were evaluated at admission by a stroke neurologist. The data of this study are available from the corresponding author upon reasonable request.

All patients with AIS admitted to our study underwent EVT from January 2013 to June 2021 were selected in the present study. Eligibility criteria for this study were (1) AIS due to a proximal large artery occlusion and treated with MT, (2) mTICI ≥2b after recanalization treatment and modified Rankin Scale (mRS) ≥3 at 3 months, and (3) available and credible information about clinical data and function outcomes.

### 2.2 Data collection

The following data from the database were obtained: age, gender, body mass index, medical history associated with cerebrovascular diseases risk factors, the location of occluded artery, time from onset to puncture and recanalization, National Institutes of Health Stroke Scale (NIHSS), Alberta Stroke Program Early CT Score (ASPECTS) or posterior circulation Alberta Stroke Program Early Computed Tomography Score (pc‐ASPECTS) at admission, intravenous thrombolysis, time from onset to admission, time from onset to puncture, time from onset to reperfusion, blood pressure, emergency laboratory tests, blood pressure, etiology according to the Trial of Org 10,172 in Acute Stroke Treatment (TOAST), operation details, symptomatic intracerebral hemorrhage, global outcomes at 3 months, and long‐term assessed by mRS. All the imaging data were assessed independently by two experienced radiologists not knowing the clinical information of patients. They were demanded to reach an agreement if there was a disagreement.

Clinical outcomes were evaluated at 90 days and beyond for long‐term outcome determination. Long‐term outcomes and survival status were confirmed via standardized telephone interviews or scheduled clinical visits by an investigator blinded to the details of the procedure.

### 2.3 Endpoints assessment

The primary outcome was the score of mRS at the time of the latest follow‐up. The secondary outcomes were: (1) risk factors for long‐term unfavorable outcomes; (2) risk factors for long‐term functional dependence; (3) any cause of death; (4) Kaplan–Meier curve for the long‐term survival probability. A score of 3–6 represented functional dependence, a score of 4–6 represented unfavorable outcomes, and a score of 6 represented death.

### 2.4 Statistical methods

First, we analyzed the baseline characteristics of patients with and without FR. For continuous variables, mean ± standard deviation (SD) or median and interquartile range (IQR) were presented depending on whether it fits a normal distribution. *T*‐test or Mann–Whitney *U*‐tests were exerted to find out differences between groups. For categorical variables, frequency or percentage was performed to describe data, and via the Chi‐square or Fisher's exact test when comparing between groups as appropriate.

Second, we analyzed the data of patients with long‐term outcomes. A favorable outcome at long‐term follow‐up was defined as mRS score of 0–3 and an unfavorable outcome (including death and mRS score of 4–5). Kaplan–Meier survival curves were made with the Prism 8.0 statistical program (GraphPad Software) to indicate the long‐term survival probability of AIS patients with FR. In the univariate logistic regression analysis, demographics, vascular risk, factors, premorbid mRS, National Institutes of Health Stroke Scale (NIHSS), systolic blood pressure (SBP), diastolic blood pressure (DBP), ASPECTS at baseline, intravenous thrombolysis, posterior circulation infarct, TOAST, general anesthesia, aspiration alone, stent thrombectomy alone, balloon dilation alone, two or more thrombectomy measures, symptomatic intracerebral hemorrhage (sICH), decompressive craniectomy, and use of edaravone were included. After the collinearity test, in the multiple logistic regression mode, we further adjusted the factors with *p* value <0.1 in univariable logistic regression.

A two‐sided *p* value <0.05 manifested statistically significant differences and all statistical analyses were conducted using IBM SPSS Statistics 26 (IBM Corp, Armonk, NY, USA).

## 3 RESULTS

A total of 960 consecutive ischemic stroke patients underwent EVT were enrolled in our observational cohort from January 2013 to June 2021. Recanalization of the occlusion large vessel was achieved in 850 patients, 37 of them were lost to follow‐up at 3 months. In total, 333 patients achieved effective recanalization, while 480 patients obtained FR. And 22 patients with ineffective recanalization were lost during long‐term follow‐up. Finally, 458 patients with ineffective recanalization were included in the long‐term prognostic analysis.

### 3.1 Baseline characteristics

Of the 480 AIS patients with FR admitted in our study, the mean age was 66.0 years (IQR, 58.0–76.0), and 315 patients (65.6%) were male. Patients with FR were older (66.0 vs. 63.0, *p* < 0.001), had history of hypertension (73.3% vs. 66.1%, *p* = 0.026), coronary artery disease (24.0% vs. 15.9%, *p* = 0.005), atrial fibrillation (35.8% vs. 28.8%, *p* = 0.037), diabetes melltius (33.8% vs. 21.0%, *p* < 0.001), hyperlipidemia (67.9% vs. 42.0%, *p* < 0.001), ischemic stroke (30.8% vs. 20.4%, *p* = 0.001), higher premorbid mRS (0 vs. 0, *p* = 0.043), NIHSS (13.0 vs. 17.0, *p* < 0.001), SBP (150.0 vs. 143.0, *p* < 0.001), DBP (84.0 vs. 80.0, *p* = 0.048), lower ASPECTS (8.0 vs. 9.0, *p* < 0.001) and posterior circulation infarct (32.5% vs. 22.8%, *p* = 0.003). The demographic and clinical characteristics of both groups are summarized in Table 1.

**TABLE 1:** Comparison of clinical characteristics between patients with and without FR.

	Without FR *N* = 333	With FR *N* = 480	*p* Value
Demographics			
Age	63.00 (53.0–70.0)	66.0 (58.0–76.0)	<0.001
Male	257 (77.2)	315 (65.6)	<0.001
BMI	25.1 (23.0–27.5)	25.0 (22.6–27.5)	0.867
Vascular risk factors			
Hypertension	220 (66.1)	352 (73.3)	0.026
Coronary artery disease	53 (15.9)	115 (24.0)	0.005
Atrial fibrillation	96 (28.8)	172 (35.8)	0.037
Diabetes melltius	70 (21.0)	162 (33.8)	<0.001
Hyperlipidemia	140 (42.0)	326 (67.9)	<0.001
History of ICH	5 (1.5)	6 (1.3)	0.760
History of ischemic stroke	68 (20.4)	148 (30.8)	0.001
Alcoholism	131 (39.3)	133 (27.7)	<0.001
Smoking	151 (45.3)	168 (35.0)	0.003
Clinical features			
Premorbid mRS	0 (0–0)	0 (0–0)	0.043
NIHSS	13.0 (10.0–17.0)	17.0 (13.0–24.5)	<0.001
ASPECTS	9.0 (8.0–10.0)	8.0 (7.0–10.0)	<0.001
Treatment with IV alteplase	112 (33.6)	160 (33.3)	0.929
Time intervals			
OTD time	270.0 (153.0–436.0)	267.0 (160.0–405.0)	0.737
OTP time	420.0 (314.0–573.0)	380.0 (285.0–535.0)	0.940
OTR time	472.0 (377.0–622.0)	460.0 (345.0–599.0)	0.404
SBP	143.0 (130.0–156.0)	150.0 (135.0–167.0)	<0.001
DBP	80.0 (72.0–90.0)	84.0 (77.0–90.5)	0.048
Posterior circulation infarct	76 (22.8)	156 (32.5)	0.003
TOAST			
Large artery atherosclerosis	210 (63.1)	275 (57.3)	0.100
Cardioembolism	104 (31.2)	184 (38.3)	
Other or undetermined	19 (5.7)	21 (4.4)	

Abbreviations: ASPECTS, Alberta Stroke Program Early CT score; BMI, body mass index; DBP, diastolic blood pressure; IV, intravenous injection; NIHSS, National Institutes of Health Stroke Scale; OTD, onset to door; OTP, onset to puncture; OTR, onset to perfusion; SBP, systolic blood pressure; sICH, symptomatic intracerebral hemorrhage; TOAST: Trial of Org 10,172 in Acute Stroke Treatment.

### 3.2 Clinical outcomes

We found an effective recanalization rate of 41% with endovascular therapy at our stroke center. With a median observation period of 7 months (IQR, 3–17; range, 1–47), 136 patients (29.7%) had a favorable outcome and 70 patients (15.3%) achieved functional independence. The median mRS score at long‐term in patients with FR was 5 (IQR, 3–6).

**FIGURE 1:**
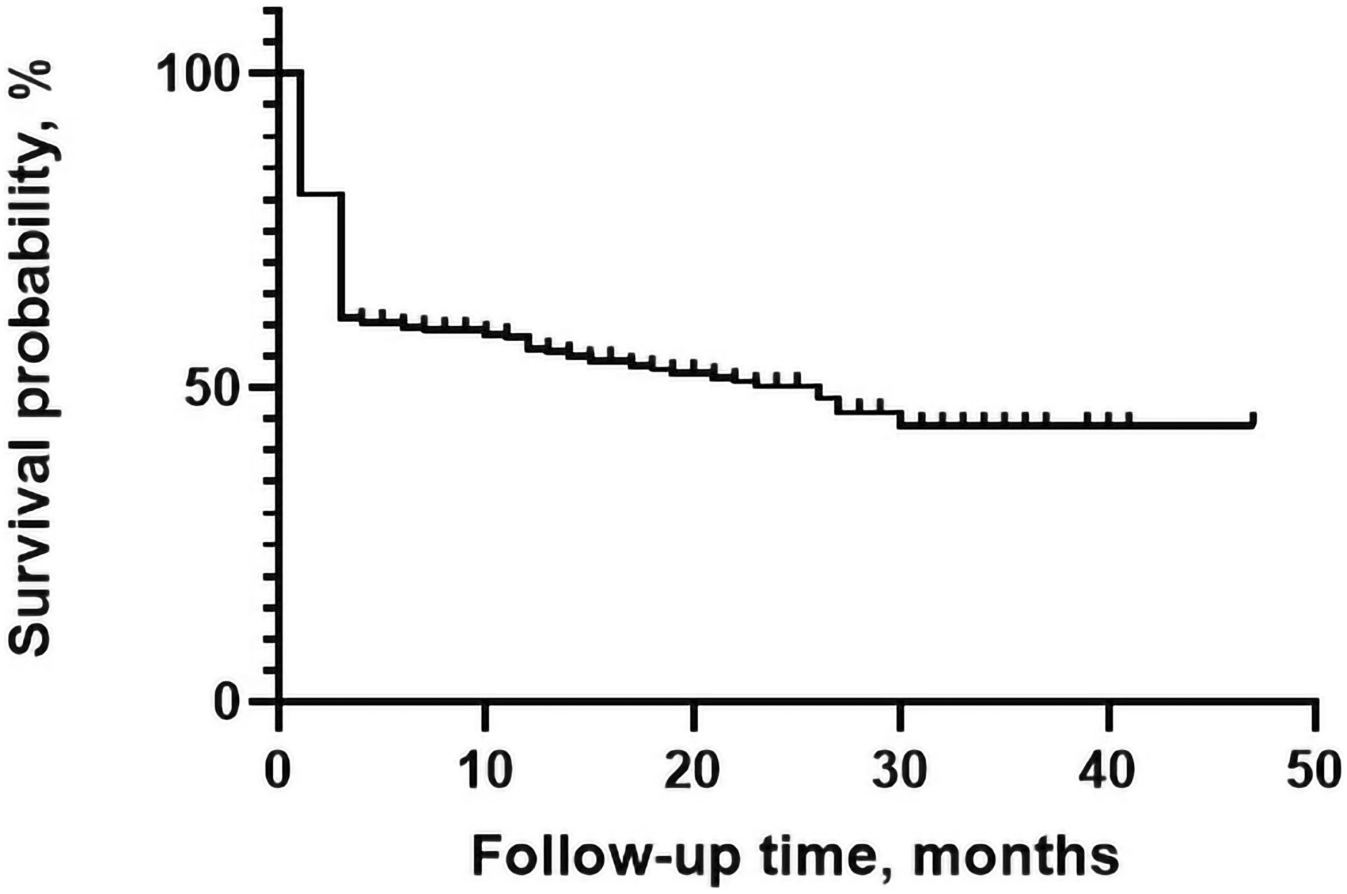
Kaplan–Meier estimate of survival probability in patients with FR.

Among all patients, 212 subjects (46.3%) died at the time of follow‐up. The time of median survival is 26 months. 175 (38.2%) patients died within 3 months after EVT. Kaplan‐Merier Curve manifests a survival probability of 56.4% at 1 year, 50.4% at 2 years, and 44.2% at 47 months after stroke in patients underwent EVT. Details of the curve are shown in Figure 1.

### 3.3 Prognostic factors

We found confounding factors that may affect the clinical outcome of patients by univariate logistic regression analysis (Data not shown). After adjustment for potential confounders, multivariate regression analysis demonstrated that age (OR, 1.058; 95% CI, 1.034–1.082; *p* < 0.001), premorbid mRS (OR, 1.864; 95% CI, 1.100–3.160; *p* = 0.021), NIHSS (OR, 1.043; 95% CI, 1.009–1.078; *p* = 0.013), posterior circulation infarct (OR, 3.303; 95% CI, 1.688–6.462; *p* < 0.001), general anesthesia (OR, 2.005; 95% CI, 1.190–3.380; *p* = 0.009), sICH (OR, 2.790; 95% CI, 1.347–5.780; *p* = 0.006), decompressive craniectomy (OR, 21.035; 95% CI, 4.608–96.024; *p* < 0.001) are associated with unfavorable outcomes in long‐term.

Factors associated with functional dependence identified by the multivariable regression analysis included age (OR, 1.048; 95% CI, 1.021–1.076; *p* < 0.001), NIHSS (OR, 1.056; 95% CI, 1.020–1.093; *p* = 0.002), and decompressive craniectomy (OR, 4.743; 95% CI, 1.054–21.348; *p* = 0.043).

And as for mortality, age (OR, 1.022; 95% CI, 1.002–1.042; *p* = 0.028), premorbid mRS (OR, 1.555; 95% CI, 1.112–2.173; *p* = 0.010), NIHSS (OR, 1.555; 95% CI, 1.112–2.173; *p* = 0.001), general anesthesia (OR, 1.556; 95% CI, 1.022–2.367; *p* = 0.039), and sICH (OR, 2.175; 95% CI, 1.272–3.718; *p* = 0.005) are prognostic factors of death at long‐term follow‐up. Data on the multivariable logistic regression analysis are indicated in Tables 2, 3, 4.

**TABLE 2:** Multivariate analysis of long‐term unfavorable outcomes in patients with FR.

Variates	OR	95% CI	*p* Value
Age	1.058	1.034–1.082	<0.001
Hypertension	0.968	0.548–1.710	0.911
Coronary artery disease	1.488	0.810–2.733	0.201
History of ischemic stroke	1.308	0.730–2.346	0.367
Premorbid mRS	1.864	1.100–3.160	0.021
NIHSS	1.043	1.009–1.078	0.013
SBP	1.009	0.999–1.020	0.077
Posterior circulation infarct	3.303	1.688–6.462	<0.001
TOAST			
Large artery atherosclerosis	Reference	Reference	0.748
Cardioembolism	1.241	0.699–2.202	0.461
Other or undetermined	1.237	0.347–4.412	0.743
General anesthesia	2.005	1.190–3.380	0.009
Decompressive craniectomy	21.035	4.608–96.024	<0.001
sICH	2.790	1.347–5.780	0.006

Abbreviations: ASPECTS, Alberta Stroke Program Early CT score; NIHSS, National Institutes of Health Stroke Scale; SBP, systolic blood pressure; sICH, symptomatic intracerebral hemorrhage; TOAST, Trial of Org 10,172 in Acute Stroke Treatment.

**TABLE 3:** Multivariate analysis of long‐term dependence outcomes in patients with FR.

Variates	OR	95% CI	*p* Value
Age	1.048	1.021–1.076	<0.001
Coronary artery disease	2.241	0.960–5.233	0.062
Atrial fibrillation	0.517	0.159–1.685	0.274
Smoking	0.855	0.410–1.823	0.686
Alcoholism	0.824	0.381–1.781	0.623
Premorbid mRS	2.380	0.996–5.688	0.051
NIHSS	1.056	1.020–1.093	0.002
TOAST			
Large artery atherosclerosis	Reference	Reference	0.502
Cardioembolism	1.900	0.632–5.715	0.253
Other or undetermined	1.451	0.350–6.004	0.608
SBP	1.011	0.999–1.023	0.066
General anesthesia	1.542	0.838–2.838	0.164
Decompressive craniectomy	4.743	1.054–21.348	0.043
sICH	1.941	0.802–4.699	0.141

Abbreviations: ASPECTS, Alberta Stroke Program Early CT score; NIHSS, National Institutes of Health Stroke Scale; OTD, onset to door; OTP, onset to puncture; OTR, onset to perfusion; SBP, systolic blood pressure; sICH, symptomatic intracerebral hemorrhage; TOAST, Trial of Org 10,172 in Acute Stroke Treatment.

**TABLE 4:** Multivariate analysis of long‐term mortality in patients with FR.

Variates	OR	95% CI	*p* Value
Age	1.022	1.002–1.042	0.028
Coronary artery disease	1.420	0.875–2.303	0.155
Atrial fibrillation	1.095	0.524–2.287	0.810
History of ischemic stroke	0.872	0.536–1.419	0.582
Premorbid mRS	1.555	1.112–2.173	0.010
NIHSS	1.046	1.019–1.074	0.001
SBP	1.008	0.999–1.016	0.071
Posterior circulation infarct	1.462	0.839–2.546	0.180
TOAST			
Large artery atherosclerosis	Reference	Reference	0.724
Cardioembolism	1.214	0.592–2.490	0.597
Other or undetermined	1.478	0.501–4.366	0.479
General anesthesia	1.556	1.022–2.367	0.039
Additional intra‐arterial thrombolysis	2.214	0.843–5.814	0.107
sICH	2.175	1.272–3.718	0.005

Abbreviations: ASPECTS, Alberta Stroke Program Early CT score; NIHSS, National Institutes of Health Stroke Scale; OTD, onset to door; OTP, onset to puncture; OTR, onset to perfusion; SBP, systolic blood pressure; sICH, symptomatic intracerebral hemorrhage; TOAST, Trial of Org 10,172 in Acute Stroke Treatment.

## 4 DISCUSSION

To date, this is the first study to report the long‐term clinical outcome in patients with FR after ischemic stroke. We found that 29.7% of patients achieved a favorable outcome and 15.3% achieved functional independence at the long‐term follow‐up, and 56.4% of patients with FR survived at 12 months, 50.4% at 24 months, and 44.2% at 47 months after stroke. Furthermore, age, premorbid mRS, NIHSS, posterior circulation infarct, general anesthesia, sICH, and decompressive craniectomy are related to unfavorable outcomes in long term.

The HERMES study suggests that, despite good vascularization, one‐third of patients do not benefit from treatment, known as “futile recanalization”. The mechanisms of FR remain uncertain yet. Several potential mechanisms have been suggested, including poor collateral circulation, hemorrhagic transformation, early arterial reocclusion, large hypoperfused volumes, no‐reflow, and impaired cerebral autoregulation.^7^ A recent meta‐analysis included 12 EVT clinical studies showed that the rate of FR after EVT was 32.4%–56.7% in patients with anterior circulation occlusion^8^ and 46% in patients with posterior circulation occlusion. Similar to these previous studies, our study showed the rate of FR was 59%.

Therefore, recently published articles increasingly focus on the influencing factors of ineffective recanalization. In terms of clinical indicators, recent meta‐analyses have indicated that older age, female, admitted SBP, diabetes, atrial fibrillation, occlusive site, without IVT, and sICH after EVT are risk factors for FR.^8^ For blood biomarkers, IL‐6, C‐reactive protein (CRP), ADAMTS‐13 has been reported to be significantly associated with FR.^9^ With regard to image markers, white matter disease and cerebral atrophy are related to FR.^10^, ^11^ However, these studies focused on the short‐term prognosis of patients and did not observe and follow up on long‐term survival prognosis and influencing factors of these patients.

Most previous studies have focused on long‐term outcomes or mortality after thrombectomy but have not paid close attention to patients with FR. MR CLEAN reported their mortality and long‐term favorable outcome (a modified Rankin scale score of 0–3) of 30.40% and 55.1% respectively at 2‐year.^12^ And REVASCAT showed a favorable outcome (a modified Rankin scale score of 0–3) of 65% at 1 year.^13^ Zhao et al. found that 53% of subjects underwent EVT were functional independence and 28% had died.^14^ At 5 years of follow‐up, Gong et al. showed that the mortality rate of patients receiving thrombectomy was 48.2%, and the rate of favorable outcome was 46.9%.^15^ However, the above studies only analyzed the long‐term outcomes of patients with anterior circulation large vessel occlusion. In recent years, studies involving anterior and posterior stroke have shown that the proportion of patients with favorable outcomes after thrombectomy has ranged from 36% to 69.1%, and mortality has ranged from 12.7% to 26% at 1 year.^16^, ^17^ In our study, it is obvious that the long‐term mortality and unfavorable outcome rates were high because of the inclusion of patients with posterior circulation infarction and the special focus on patients with FR.

As for the factors influencing the long‐term outcome after EVT, studies have shown that age, pre‐stroke functional status, posterior circulation stroke, recanalization grading, ASPECTS, NIHSS, postoperative sICH, and mRS at 3 months may be the significant predictors.^17^, ^18^, ^19^, ^20^ In addition to the influence factors similar to previous studies, general anesthesia, sICH, and decompressive craniectomy were found to be significant predictors of unfavorable outcomes in our study. General anesthesia and sICH were also observed to be associated with mortality in this study compared to the previous reports. The effect of anesthesia on the prognosis of patients underwent EVT is still controversial. Previous studies have indicated that the advantage of general anesthesia for patients lies in reducing patient agitation, thus improving surgical accuracy and reperfusion rate.^21^ However, in patients with FR, general anesthesia is associated with unfavorable long‐term outcomes, possibly due to intervention delay, hypotensive episodes, and difficulties in respiratory management. This result needs further consideration and verification. In addition, we found no association between aspiration alone, stent thrombectomy alone, balloon dilation alone, or two or more thrombectomy measures and long‐term prognosis of patients with FR. Therefore, the postoperative management of the blood pressure and so on to prevent the occurrence of sICH may bring more benefits to the long‐term prognosis of the patients, even if the prognosis was unfavorable at 90 days.

There were several limitations to consider. First, this was a partially retrospective study of a single‐academic center, although it reflected real‐world experience. Second, despite the multivariable regression analysis was used to adjust for relevant variables, the possibility of potential confounding factors remained, such as rehabilitation or medication after discharge and so on. However, not all survived subjects returned for in‐person clinical visits, so telephone return visits will cause certain bias of these data analysis. Third, due to the limited storage time of partial image data, the image data were incomplete and could not be included in the analysis more comprehensively.

## 5 CONCLUSIONS

In conclusion, among patients with FR undergoing thrombectomy, the 1‐year survival rate is 56.4%, and the 2‐year survival rate is 50.4%. Age, premorbid mRS, NIHSS, posterior circulation infarct, general anesthesia, sICH, and decompressive craniectomy are independently associated with long‐term unfavorable outcome. It is urgent to pay attention to the ineffective recanalization after endovascular therapy in patients with ischemic stroke. These findings suggest that, on the one hand, the pathogenesis of FR has not been fully clarified and a unified prediction scheme is lacking, on the other hand, this reveals the long‐term survival and prognosis of the patients with FR in the real world, which provides a certain reference for the long‐term treatment guidance and prognosis prediction of these patients.

## AUTHOR CONTRIBUTIONS

Mengke Zhang, Ruiwen Che, Xunming Ji designed the study. Jiali Xu, Wenting Guo, Xi Chen, Wenbo Zhao, Changhong Ren, Milan Jia collected the data. Mengke Zhang analyzed the data. Mengke Zhang drafted the manuscript. Ruiwen Che and Xunming Ji revised the manuscript.

## FUNDING INFORMATION

This study was funded by the National Natural Science Foundation of China (No. 82001257), the National Natural Science Foundation of China (No. 82027802), and the Natural Science Foundation of China (No. 81971114).

## CONFLICT OF INTEREST STATEMENT

The authors claim no disclosures in this manuscript.

## Data Availability

The data is available on request from the corresponding author if there is a reasonable demand.
